# “…This Has to Do With My Identity. And I Don't Want to Make it Totally Transparent.” Identity Relevance in the Attitudes of Affected People and Laypersons to the Handling of High-Throughput Genomic Data

**DOI:** 10.3389/fsoc.2020.532357

**Published:** 2020-12-01

**Authors:** Alexander Urban

**Affiliations:** ^1^Department of Medical Ethics and History of Medicine, University Medical Center Göttingen, Göttingen, Germany; ^2^Faculty of Social Sciences, Georg-August-University Göttingen, Göttingen, Germany

**Keywords:** qualitative social research, identity, medical sociology, genomic information, genomic high-throughput sequencing, identity construction

## Abstract

With the establishment of genome sequencing, the influence of genomic information on self-understanding and identity construction has become increasingly important. New sequencing methods far exceed previous genetic tests in terms of scope and quantity. Despite theoretical approaches, however, there are few empirical findings on the identity-relevant influence of genomic information. The present study examines genomic information's identity-relevant influences and considers whether developments in the field of genome sequencing may generate problems that are not yet addressed by existing identity concepts based on traditional genetic tests. The study is based on 10 partially standardized interviews with personally affected persons and four focus groups with medical laypersons as representatives of the public, which were evaluated on the basis of qualitative content analysis. As a result, this paper presents five thematic areas with identity-relevant references within subjective attitudes toward the handling of genomic information, and also derives two basic identity concepts. The results indicate that the lay discourse is still strongly based on older debates about genetic testing and that the view on the complexity of genomic information established in the scientific context has thus far no influence on the perspectives either of those affected or laypersons.

## Introduction

With every medical-technical quantum leap in genetic diagnostic technologies, the debate about the social impact of genetic information and its influence on people's self-understanding and identity is rekindled. Scientists from the Genom Austria project, for example, see the genome as a comprehensive source of information about individual health aspects (Taschwer, 2015); the project is part of Harvard Medical School's global Personal Genome Project under the leadership of George Church (Open Humans Foundation, 2020). The Personal Genome Project's aim is to sensitize society to the handling of genomic information, explicitly emphasizing the individuality and resulting identifiability of the genome. This shift in perspective from genes to genome leads to the question of what role genomic information plays in self-conception.

The present article therefore addresses the influence of genomic information on the identity construction and self-understanding of affected persons within the framework of the new technical possibilities of genomic high-throughput sequencing (GHS). This technology includes devices and procedures that enable ever more time-saving and cost-effective analyses of the entire human genome, or large parts of it (Flores et al., 2013; Bettecken et al., 2014; Müller-Röber et al., 2015). In contrast to conventional genetic tests, the technologies that are close to clinical implementation generate vast amounts of genomic information, often with unclear validity and no evidence of its practical effects (Guttmacher et al., 2010).

The debate as to the scientific and social implications of genetic testing and diagnostics has been ongoing for several decades (Löbsack, 1985; National Bioethics Advisory Commission, 1999; Feuerstein and Kollek, 2001; Waldschmidt, 2003). Sociological approaches to genetic identity often address the fact that subjective interpretations arise from the numerical risk data and results of genetic tests. These subjective interpretations, in turn, are based on personal and cultural beliefs as well as socially shared values and may therefore have a strong influence on self-understanding (Wüstner, 2001; Klitzman, 2009, 2012). This has resulted in various identity models: from self-understanding as a deficient based on identified susceptibilities or performance deficits (Feuerstein and Kollek, 2001) to new forms of identity conceived through the influence of genetic knowledge and closely linked to genetic responsibility (Novas and Rose, 2000). With the emergence of GHS in the last 15 years and the resulting surge in volume of information at declining costs, the theoretical approaches are increasingly shifting from a genetic to a genomic identity (Zwart, 2007, 2009; Klitzman, 2009), and the growing societal relevance of the consideration of identity under the influence of genomic information is gaining attention. This is reflected, among other areas, in the economic use of genomic data for the assessment of individual health aspects in the area of “direct-to-consumer genetic testing” (Curnutte and Testa, 2012; Gollust et al., 2017; Roberts et al., 2017; Vayena and Blasimme, 2018).

Thus far, only theoretical assumptions and limited empirical insights concerning the influence of genomic information on self-understanding and identity construction have been available (Zwart, 2007, 2009). In order to add to the body of empirical research on this subject, this paper will investigate to what extent genomic information has an influence relevant to identity. It is also important how this information is understood and described by affected and lay people and whether possible identity concepts can be derived from these descriptions. As a side issue, this paper further seeks to determine whether technological development can also induce new problems for identity construction that are not yet covered by identity-related theoretical approaches based on genetic information.

In order to investigate these questions, ten partially standardized interviews with personally affected persons and four focus groups with medical laypersons as representatives of the public were conducted. The material was analyzed on the basis of qualitative content analysis (Mayring, 2015). The results show five thematic areas in which identity-relevant references could be identified among subjective attitudes and evaluations, on addressing genomic information and GHS; from these areas, two basic interviewee identity concepts were derived. The results are discussed against the background of the current debate, demonstrating that the lay discourse is still very much based on older debates about genetic testing.

## Theoretical Background: Genes, Genome and Identity

The debate on the societal impact of genetic information is influenced by a variety of elements. One area of controversy is the handling of probabilistic information, as used in conventional genetic testing. Affected persons receive probabilistic information on certain disease risks, which are relative statements that contribute to the blurring of previously delimitable categories of health and disease (Lemke, 2006; Kollek and Lemke, 2008). Such phenomena can also be described with clear results in relation to monogenetic diseases such as Huntington's chorea, which appear only in later adulthood but can affix the label of “ill person” to the self-perception of those affected long before the first occurrence of disease symptoms (Wüstner, 2002, p. 247). Despite the absence of clinical or physical signs of late-manifesting disease, the results may induce psychological stress in patients. In the field of genetic diagnostics, genetic risk information in connection with temporal dimensions plays an important role, influencing the lives of affected persons, their families and their self-image accordingly (Atkinson et al., 2013).

At the same time, the establishment of GHS has been accompanied by increased uncertainty that poses major practical, social and ethical challenges for all groups involved. Vast amounts of data, unclear complex relationships and difficulties in data protection and the handling of additional findings^1^—as well as findings of varying quality and the challenging interpretation of statistical results—are some of the challenges associated with the technologies that are generally referred to as “next generation sequencing” (NGS). Another typical feature of GHS is its constantly growing IT- and data-based application area (Umbach et al., 2016), which extends from research and clinical application in the fields of oncology, psychiatry, epidemiology and neonatology to new approaches in individualized medicine (Schuster, 2007; Majewski et al., 2011; Desai and Jere, 2012; Schrijver et al., 2012; Biesecker and Peay, 2013; Neveling and Hoischen, 2014). The application of genomic sequencing techniques has led to increased knowledge of the complexity of genetic relationships and thus to a changed understanding of causal relationships (Zwart, 2007). This, in turn, is reflected in a new research paradigm that has also influenced many cultural and social science disciplines (Zwart, 2009). In the area of genome sequencing, the classical paradigm of science—conscious simplification in order to establish verifiability in models—has been replaced, leading to a “shift from genetic determinism to understanding complexity” (Zwart, 2007, p. 194). In this context, subjective conceptions of genetic and genomic information are becoming increasingly important (Trinidad et al., 2010; Sapp et al., 2014; Nelson et al., 2016).

### Identity in Traditional Genetic Diagnostics and Genome Sequencing

The question of the influence of genetic information on self-understanding and identity construction has thus far been raised several times in debates on genetic diagnostic possibilities, and many actual theoretical approaches to identity are based on scientific debates in ethical and sociological research on genetic testing and reproductive medicine applications. Duden and Samerski (2006) claim that the dissemination of genetic tests turns the body into a gene carrier and a statistical profile, revealing it to be a danger to the self. Novas and Rose (2000), meanwhile, emphasize that personality reconstitutions occur alongside new life strategies: apart from monogenetic diseases, disease risks based on genetic information do not represent a fixed fate, and new relationships to the self and one's own future may thus be created. Even before the establishment of GHS, Chadwick (2003) stressed that genes can be associated with ideas about our own identities in a deeper sense and through a variety of concepts—from extremely reductionist views that regard genetic information as determining identity, to those that see in humans far more than the sum of their genetic information and emphasize imprinting via experiences and the social environment. Chadwick notes that collective identities must also be taken into account, since individuals are always part of a group under whose influence decisions are made. Accordingly, genetic information can have traumatic consequences if the knowledge gained does not coincide with personal assessment, embedding in the social environment or family narratives. Identity is not constructed by an individual solely on the basis of certain facts and findings but in relation to others, embedded as a member of a number of different groups.

### Identity Relevance of Genomics

A transition to identity concepts that consider genomic information is marked by Zeiler (2007) approach, which distinguishes identity according to its relationship to genetic or genomic information and describes how different philosophical notions of identity can be combined into a single, multi-layered concept. From this perspective, numerical, qualitative, personal, genetic and competing concepts of genomic identity must be included and considered over time. Zeiler characterizes identity as an entity comprising several layers, which at different times have different levels of importance to identity and self-understanding. This complex approach takes into account the genome's far more comprehensive information content, along with the fact that identity is not based solely on a single layer, namely genomic information (Hauskeller, 2004; Zeiler, 2007). The blurring of the boundaries between health and illness for the aforementioned “patient in waiting” also remains an important aspect of identity (Timmermans and Buchbinder, 2010) but does not represent a comprehensive identity concept, as it is limited to only part of the problem of dealing with genomic information. Within the social science and humanities debate, there is also a fundamental conviction that genomic information has an impact on the self-understanding and identity construction of affected people (Zwart, 2007, 2009). According to Atkinson et al. (2006), this information can change our self-conception as social actors and our perception of biographical development and personal stability.

As early as the first decade of the 2000s, researchers assumed that genomic information would be used as a source of self-knowledge in the future (Zwart, 2007). In the meantime, studies have demonstrated the public's personal interest in genomic information derived from scientific and medical testing (O'Daniel and Haga, 2011), including information about one's own genetic profile (McGowan et al., 2013) or data about familial pre-dispositions, such as depression (Wilde et al., 2009). According to Zwart (2009), the expansion of the technical and scientific possibilities of GHS has been accompanied by a “bioinformatization” of human life, which, due to its rapid establishment, has had a far stronger influence on the understanding of identity-forming processes and the consideration of the self than conventional genetic testing methods.

Nevertheless, the question of how genomic information influences everyday understanding of the self, as well as interpretation and representation of the self, remains an important field of social science research. Analyses are therefore needed “that can only be achieved with empirical data to draw out the complex interrelationships between identities, technologies, self-knowledge, and the future” (Fishman and McGowan, 2014, p. 38).

### Theoretical Aspects of Identity

The underlying concept of identity is to be understood as an ongoing process based on interaction, attribution and self-attribution. The sociological view taken here is based on Herbert Mead's (1934) understanding of identity, according to which identity does not emerge from the individual by itself, but is a reflexive process under mutual influence of the members of a social group within a society. It is important to distinguish identity from psychological understanding: identity is not understood here as a subjective, isolated and independent element to which only the organism has access—this, according to Mead (1934, p. 164), is rather described by the concept of consciousness. Reflexivity as the basis of the understanding of identity is further shaped by Anthony Giddens (1991) theory, which describes identity as created through negotiation, attribution and self-labeling using specific narratives, information and personal experiences.

### Aspects of the Empirical Analysis of Identity-Relevant Statements

How can genomic information become identity relevant? One can speak of identity relevance when interviewees perform an act of social construction in their self-descriptions by locating themselves socially in relation to a specific object (in this case genomic information) and creating a match between their subjective insides and the social outsides (Keupp, 2018). In descriptions of personal attitudes and opinions with regard to GHS technologies and information, such social constructions and locations are often made. As the interviewees speak about themselves, they become objects to themselves—a basic pre-requisite for the construction of identity. According to Mead (1934, p. 142), the portrayal of subjective attitudes and opinions becomes relevant to identity when respondents themselves become objects, react to themselves and relate to something outside of themselves. In this way, identity relevance in the description of attitudes and personal experiences around genomic information can be determined through an interpretation process. Within the framework of this social construction and location, genomic information can influence identity formation. The extent of this influence can also be derived from Mead's theory:

“*We divide ourselves up in all sorts of different selves with reference to our acquaintances. We discuss politics with one and religion with another. There are all sorts of different selves answering to all sorts of different social reactions”* (Mead, 1934, p. 142).

From this, the thesis can be derived that identity will not normally be formed entirely through genomic information but that parts of self-representation within communication with others will be influenced by it. This also leaves room for integrating the approach of Zeiler's layered model of identity (Zeiler, 2007). Using the theoretical approach outlined above, identity-relevant aspects can be inductively recorded and described in attitudes to genome sequencing and genomic information. The result of this interpretative process is presented in the results section. Despite the theoretical approaches developed thus far and an intensifying debate, there is a lack of empirical stocktaking, especially of identity-relevant aspects of genomic information. Above all, the inclusion of both affected and lay perspectives as well as corresponding subjective attitudes and opinions denotes an underrepresented field to which this article is intended to contribute.

## Methods

The present study's methods of recruitment, conducting of interviews and focusgroups and analysis of the material are presented in this section. All important aspects for reporting qualitative research results have been taken into account based on the consolidated quality criteria of qualitative research (COREQ checklist) (Tong et al., 2007).

In order to survey the attitudes of personally affected persons and laypersons in dealing with GHS, two methodological approaches were combined: partially standardized interviews were conducted with personally affected persons, and the attitudes of medical laypersons were surveyed in focus groups. The advantage of combining the methods lies in the use of different influences in each conversation situation. Individual interviews release interlocutors from their accustomed environment and enable the capturing of attitudes and statements without direct group pressure. This method appeared to be more suitable for the survey of experienced or personally affected persons, since in a remote interview situation, the focus could be placed on subjective attitudes and opinions about the personal experiences of those concerned. Focus groups, by contrast, allow only for attitudes and opinions that are formulated in the situational group discussion context (Halbmayer and Salat, 2011). For the survey of medical laypersons, the aim was to map public discourses, as well as to record the use of arguments and attitudes in connection with the discussion about GHS and the use of genomic information. The aim in combining the two methods was, when possible, to compare and analyze different statements and generally shared views in both survey contexts and to determine identity-relevant references in the survey material. This approach has been established in applied empirical social research (Raz and Schicktanz, 2009; Daack-Hirsch et al., 2013; Nelson et al., 2016). The procedure is explained below.

### Interviews

From June 2015 to October 2016, a total of ten interviews were conducted with personally affected persons who had their own experiences with GHS. The search was for persons who, as patients or participants in relevant studies, came into contact with corresponding methods of GHS or whose genomes were sequenced for diagnostic or research purposes. Recruitment proved difficult, as there were no major studies using genomic high-throughput sequencing technology at the time and contact with potential study participants had to be established through study nurses. Due to these challenge, people were initially recruited who had already had personal experience with genetic testing in order to ascertain their subjective perspectives. There were two recruitment pools in the medical setting: Six persons who had initially participated in a genome-wide association study for the investigation of bipolar disorders were recruited via flyers and notices in a psychiatric area of a university hospital; of these, four individuals had come into contact with GHS as part of a control group rather than as patients. The second area was limited to a surgical department of a university hospital that largely treated colon cancer patients; the study nurses recruited two colorectal cancer patients who had participated in a study on the cancer genome as part of their treatment.

Overall, the selection of participants was made in the spirit of purposeful sampling (Coyne, 1997; Palinkas et al., 2015), which provides for a targeted selection of participants—which, in the case of the present study, refers to the criterion of personal involvement. This is understood as an authentic or embodied experience via participation in studies that included GHS or genetic testing (Schicktanz et al., 2008). A total of eight persons from the psychiatric field and two from the oncological field were included. One of the respondents from the psychiatric context had received a diagnosis based on the sequencing, and another based on a genetic test. The other respondents had provided their genomic or genetic information for purely scientific purposes and had not received a diagnosis at the time of the interviews. The participants consisted of six academics, two people with intermediate educational qualifications and two people from the working-class milieu. The participants were between 31 and 64 years old. The interviews were conducted solely by the author (male, M.A. in sociology) as a junior scientist within a research project (see Funding), who at the time of the study had several years of experience in the use of qualitative interviews in various health care settings. The interviews were conducted at the author's workplace in a protected atmosphere and lasted between 45 and 90 min. Field notes were made after the interviews about the atmosphere and course of conversation as well as important aspects concerning the content of the conversation.

### Focus Groups

In cooperation with another research project (see Acknowledgments), seven focus groups with 43 participants representing the public were conducted between June 2016 and November 2016. Medical laypersons were recruited who had had no contact with GHS at the time of the survey; two participants, however, had already taken part in a genetic examination. The focus groups took place in four German cities (Berlin, Göttingen, Frankfurt am Main and Cologne). The participants were recruited via posters, flyers, Facebook and eBay classified advertisements. Prior to participation, participants were informed of the objectives of the study and focus group survey. The group composition was, where possible, consciously selected and mixed in terms of educational background, age, gender and socio-economic status (Coyne, 1997; Palinkas et al., 2015). The discussions took place in a sheltered setting and usually lasted between one and a half and 2 h. The participants were able to discuss the questions and their own points of view. The conversations were moderated by two scientists who, in order to exclude any suggestive influences, did not engage in the discussion (Krueger, 1998; Krueger and Casey, 2000).

### Interview Guidelines

In order to inform respondents about possible problems in dealing with the technology and its data, four different vignettes (Table 1) based on the current state of scientific knowledge regarding GHS-related ethical and social problems were produced for the partially standardized interview guidelines. The vignette provided the respondents with a stimulating starting point designed to encourage them to make further statements (Stiehler et al., 2012); moreover, the assessment of concrete vignettes is far closer to real judgement in everyday life than the answering of comparatively general and largely abstract questions (Dülmer, 2016). Distortions or suggestive clues must be avoided, and the interviewee must be able to judge freely. Four vignettes were also used in the focus groups, which, since they were developed in cooperation with another research project, also included other topics (i.e., direct-to-consumer genetic testing, use of biomarkers). This article focuses on the detailed analysis of the third vignette, while the others are analyzed and published elsewhere (Schaper et al., 2018; Wöhlke and Perry, 2019; Wöhlke et al., 2019); this selection is due to the fact that the vignettes were designed to address various ethical issues in the field of genetic and genomic information. The focused analysis took into account the general discussion process and each group dynamic. Both guidelines focused on aspects of informed consent, feedback on the findings, dealing with different qualities of outcomes, knowledge needs of stakeholders, different application contexts and data management. None of the respondents was asked about personal test results obtained in GHS or genetic testing. Both guidelines were pilot tested, revised and refined before they were applied. All of the respondents spoke of their own free will.

**Table 1:** Presentation of vignettes for interviews and focus groups.

**Vignettes/scenarios of the interviews**	**Topics**
**Scenario one** A person has her genetic make-up examined at the age of 30 because one of her parents was diagnosed with dementia. As a result of this examination, statements are made about the likelihood that she will also develop dementia in the future. It turns out that she has an 80 percent risk of developing an early-onset form of dementia within the next 10 years.	• Understanding of probabilistic information • Dealing with the likelihood of disease • Information required for a better understanding • Possible effects on the lives of those affected • Dealing with incidental findings
**Scenario two** An adult person is invited to participate in a study in which his or her entire genome is analyzed in a short period of time. A lot of data is collected that could provide information about blood group, ethnic origin and intelligence, but also about disease risks. If desired by the participant, it is possible to learn about one's entire genetic information within the framework of this study.	• Attitude to technological possibilities • Data protection and access rights • Assessment of the collection of medically irrelevant data • Digital self-monitoring and optimization
**Scenario three** An adult person has submitted a blood sample to have his or her genetic make-up analyzed. The analysis does not give rise to anything conspicuous, but the genetic information from the sample is stored indefinitely. In the future, new methods of analysis and new insights into associations between genes and diseases could lead to a situation where the sample could provide new information on disease risks.	• Assessment of permanent data storage • Data storage by different stakeholders (authorities, companies) • Long-term use and subsequent generation of personally relevant results
**Scenario four, part one** Tumors consist of cells whose genetic material is altered. Imagine a genetic examination that determines whether a planned therapy will be successful in a given patient. The therapy may have considerable side effects and significantly reduce the affected person's quality of life without having any benefit for her.	• Attitudes to stratification of patients • Differences in personal affectedness • Problems and dangers from respondents' point of view
**Scenario four, part two** As a result, it turns out that the therapy has a chance of success in about ten out of 100 people. The patient is offered a palliative treatment in order not to burden her remaining lifetime with the stresses and side effects of the therapy.	
**Vignettes/Scenarios Focus groups**	**Topics**
**Scenario three** A person (about 30 years old) is invited to take part in a medical study in which their complete genome, i.e., their complete genetic information, is also recorded and analyzed in a short time. A lot of data is collected that could provide information about blood group, ethnic origin and intelligence, but also on various hereditary diseases and disease risks.	• Attitude to generation of personally relevant results and technical possibilities • Data protection and access rights • Evaluation of the collection of medically irrelevant data • Evaluation of long-term storage • Storage by various stakeholder groups (public authorities, companies) • Long-term deployment and subsequent generation of personally relevant results

All conversations were audio recorded with tape recorders and then transcribed by an assistant. In the course of the transcription, all sensitive data, names and locations were pseudonymized. The analysis of the resulting material was conducted using qualitative content analysis according to Mayring (Mayring, 2000, 2015; Gläser and Laudel, 2009). The software Atlas.ti^TM^ was used for the coding, and the data were coded solely by the author.

All participants of the interviews and focus groups were informed in advance about the study and its data protection aspects as well as the interests of the interviewers in the research topic and the reasons for the scientific investigation. Participation could be revoked at any time without providing a reason. Two people refused to participate due to low motivation. The participants did not receive any feedback on the findings. The study and the interviews were approved by the Ethics Committee of the University Medical Center Göttingen (Ethics Proposal #16/10/14). The implementation of the focus groups was also approved in an amendment.

## Results: Identity-Relevant References to Genomic Information

The following section presents identity-relevant aspects from the interview and focus group material. These aspects are characterized by the creation of a self-reference to the topic and can be identified primarily in the context of evaluations, judgments and opinions, which are usually linked to personal experiences in the medical context or the social environment or are based on biographical passages. The relevance of identity in the interviewees' statements must therefore always be interpreted in the context of the participants' own experiences and seen as a component of social construction in the field of communication between interviewer and interviewee.

The results will be briefly summarized and then explained in more detail. On the basis of the interview and focus group material collected, five topic areas with identity-relevant references were found.

The first identity-relevant area relates to deterministic and exceptionalistic attributions regarding the expressiveness of genomic information. Some interviewees assumed that this information would determine pre-dispositions, characteristics and abilities as well as developmental potential and character traits. Above all, the interviewees addressed the availability of the most sensitive information—the transparency of one's identity and the resulting danger of interference in the private sphere along with possible stigmatization.

The second identity-relevant topic is closely linked to the use and misuse of genomic information and the associated dangers for the development, representation and construction of identity. Due to its special status, the interviewees regarded genomic information as property worth protecting, and they considered the safeguarding of privacy to be more important than any potential medical benefits. Fears that one's own fate can be controlled and predicted by genomic information were also addressed. Some interviewees further believed that assumed efforts to optimize and improve human beings threaten to deliberately interfere with self-determination and ultimately take away their identities.

The third area demonstrates the identity relevance of genomic information in the family. Two fundamental effects were discussed here: on the one hand, the threat posed by the findings to the self-image of the family and its individual members. Within this framework, information avoidance as a technique of information control becomes a strategy for protecting the family self-image from damage if the family does not agree with the statements made on the basis of the sequencing. On the other hand, participants emphasized the possibility to confirm family related health burdens. This was seen as a validation for self-understanding. The relevance of identity is clear here in the self-reference to genomic information against the background of biographically related descriptions and their confirmation and rejection. Biographical and family narratives thus become the reference point for attitudes toward the handling of genomic information.

The fourth identity-relevant area also focuses on biographical topics, but more on future lifestyles, including the uncertainties of probabilistic statements regarding possible psychological burdens and drastic changes in lifestyle. Participants saw genomic information as a possible cause for change in attitudes and lifestyles, as the current self-understanding of healthy people can be significantly impaired by focusing on test results and possible onset of disease. Even though this is not proof of permanent change in behavior, health-relevant behavior is aligned with genomic information, and an active relationship with the self and one's own future may be instigated. Self-referential information can become either more or less relevant depending on the life situation or biographical point in which those affected find themselves. Genomic information may also gain or lose relevance in terms of a layered model of identity. In phases of severe illness or old age, such information may no longer offer important insights, which further suggests that the identity relevance of genomic information is context-dependent.

In the fifth topic area, genomic information's added value to identity through the possibility of legitimation, attribution and self-attribution point to identity-relevant references. These are seen above all in the relieving function against negative attributions by third parties and in the context of external family representation in order to avoid stigmatization from the outset. Due to new diagnostic possibilities, genomic information is ascribed a positive influence, which has an identity-forming character.

Although there are many areas of overlap between these topics, they can be distinguished according to certain aspects: some are attributions of high significance and others are the resulting effects of this attribution, such as the identity gains and losses described. The third possibility of differentiation exists in the dangers and possibilities posed by genomic information, which become clear in the context of confirmation or destruction of personal and family identity. This does not, however, represent a conclusive categorization, since further research may reveal additional identity-relevant aspects. Concrete differences in attitude between those personally affected and laypersons could only be identified with regard to the different experience backgrounds in the assessment of the vignettes. Those personally affected increasingly seemed to rely on biographical experiences in the medical field in order to justify their attitudes and opinions, while the focus group participants, who were recruited as laypersons, tended to base their views on generally shared knowledge and opinions. Nevertheless, there were also important differences between psychiatric and cancer patients with regard to genomic information's degree of identity relevance, as well as the type of disease and its severity. This shows that generalizations can only be derived very cautiously from the respondent groups. Otherwise, due to the complexity of the topic and the low level of participant knowledge regarding the goals, backgrounds and problems of genome sequencing, there were no major differences in response behavior between the two groups.

### Attributions: Genomic Information Yields Knowledge About one's Own Identity

The material is dominated by exceptionalistic views on genomic information. These views assign genomic information a special status, since it enables statements to be made about, for example, disease risks or causes of diseases as well as physical-psychological conditions. The interviewees understood it as extremely sensitive and meaningful information that allows statements to be made about a wide range of personal characteristics, ranging from character disposition to development potential and abilities. Despite the common exceptionalist view of the relevance of this information, ideas about the determinacy of human traits based on genetic principles differed widely. The following two quotations, derived from one of the focus groups, show the potential that was attributed to genomic data:

“*And as far as I know, one can use the information from genome analysis to predict some properties of my character. I find that very bad. If such a scientist has this private information about me, he can predict how I will react to different situations. I don't want such things” (Person 29, female, FG V 25:04)*.

Here, genomic information is attributed to an extremely high significance and the prediction of individual character traits is emphasized. The deterministic assumption is that the human genome defines character traits. This attitude was observed in several of the subjects and resembles, in part, a genetic reductionism, from which perspective everything human—from behavior to physical characteristics and psychological conditions—is genetically determined. In this conception, genomic information makes visible the most personal and subjective aspects of human beings. It also allows for the prediction of a person's behavior, which poses a threat to the protection of privacy. A similar quote from a participant of another focus group shows that the collection of genomic data is associated with a vast amount of information, which makes it possible to learn everything about the person involved:

“*On the one hand, I find it very interesting that you can find out so many things. On the other hand, they know a lot about you and I don't know if I would do that, for example. Because then I think to myself: “Ok, one knows so much about me, one knows everything about my origin, one has my DNA, my data, everything, actually”” (Person 18, female, FG III 25:30)*.

Another view expressed in one of the focus group discussions was that genomic information creates a basis for comparison between a person's actual and possible development:

“*Because then maybe I'd be desperately unhappy. Because I could compare. Or, assuming I would compare now. “Girl, what possibilities have you had and what have you actually done with your life?” So. No, I wouldn't want to know. And I wouldn't take part in the study either, by the way” (Person 30, female, FG V 25:03)*.

Here too, genomic information is assigned a special status. The participant believes that it is possible to identify human development opportunities on the basis of genomic information. This view also implies that one's genetic pre-disposition does not entirely determine the human being, but only lays the foundation for development possibilities; nevertheless, it permits information about the endpoints of possible developments. In this case, the group further discussed whether it would be worth comparing such potential with actual development later on and whether the person concerned would not be frustrated if this potential had not been fulfilled. The relevance of identity is shown here above all in the attribution that genomic information makes the self and its hidden aspects visible. The following example makes clear how deeply this information affects the self from the perspective of the interviewees: A psychiatric patient describes her discomfort with the way young people, from her perspective, handle data. She relates this uneasiness to the handling of genomic information, which she describes as the most intimate type of information:

“*That's the most intimate thing. So I'm shocked—but maybe I'm also relatively old— that you can [see] with what generosity young people distribute their data on the net. […] There you see how careful you have to be with your things and I find such bare numbers as a laboratory test and just such a highly sensitive thing like the genome where so much important information is in there with each of us, that is now, no matter if it is hereditary or not, that is so highly sensitive. That must not be outside of one's own person under any circumstances” (PSY III 13:51)*.

Here, the affected person describes a kind of layer model of identity in which genomic information concerns the most intimate part of the person. This goes beyond the concept of privacy to include as an individual's most personal sphere. It is also notable that “naked” numbers, in the interviewee's view, allow a clear statement about people and again expose them to the danger of certain intimate aspects becoming visible. The quote indicates that such sensitive information can represent a part or layer of the self and has the potential to discredit one's own identity.

Among the colorectal cancer patients questioned, similar statements referring to the exceptionalistic character of genomic information can also be found. Nevertheless, this group largely provided different, mostly positive assessments of the handling of genomic information. These statements were highly dependent on the experience gained in the context of the disease and therapy. Close comparisons were frequently made with one's own disease and the associated experiences during treatment. The following example refers to the question of the influence of the genome on human characteristics and abilities. The respondent answers here by comparing genome testing to cancer therapy:

“*Well, I mean there will be something to it that everyone is composed differently and that comes from the genes. I believe that's just the way it is. I once weighed 130 kilos and lived happily until I was diagnosed with this disease. And then I did something about it, first I lost a lot of weight because of the cancer and then I had to lose weight again because of these operations. And it worked, you adjust to it and then you do it, you have to have the will to do it. And that's how it is with these genetic things: Some people are affected, […] the others are just better. One must accept that” (ONK II 22:49)*.

From these comparisons to the participants' experiences of cancer, it can be concluded that such serious illnesses have a very high influence on self-understanding. The respondent also pleads for trust in physicians when interpreting genomic information, probabilistic data or additional findings. This must be seen against his personal background, as he claims to have had a special operation for the removal of his colon cancer that was not a standard procedure:

“*You actually have to leave up to the doctors how they deal with it […]. As a lay person, you can declare your willingness to continue research or something else, but you have to leave it more or less to the doctors if they can use it, if they can extract something from it. If there*'*s no one who is responsible for it or if no one participates, it cannot go forward, and medicine lives from research” (ONK II 11:40)*.

Despite similar attributions to the significance of genomic information, a completely different influence of genomic information on the construction of identity is thus apparent in colorectal cancer patients. As the evaluation of the vignettes is always made by including one's own disease, the problem of genomic information for the self-image seems to be overlaid by the participant's identity as a cancer patient.

### Loss of Identity Due to Transparency of Sensitive Information

Similar identity-relevant references can be found in the area of the use, transmission and dissemination of genomic data. Since genomic information, from the perspective of the respondents, is particularly sensitive information that can reveal nearly everything about them, its use for scientific and commercial purposes was critically discussed by the focus groups. This critical perspective is described in the following quote from a control group participant regarding the possibility of sequencing and scientific evaluation for non-medical purposes:

“*Well, if it's anonymized. So the researchers probably need this for research, so that they have as many samples and test persons as possible. Then I think that's fine, but if my name is on it and, instead of three people, 300 people can find out everything about me through my name and then do things to me, that would be stupid. But anonymously, I think that's okay” (PSY V 14:30)*.

In emphasizing the dangers of anonymity and the protection of his identity, the relevance of identity becomes clear: the interviewee places himself in relation to the danger of complete visibility, the loss of anonymity and the fear of being unprotected against the misuse of information by others. Indeed, the anonymization of data as well as their restriction to certain usage or research purposes in publicly funded institutions was intensively addressed by the interviewees. Genomic information was regarded as property, whose access rights and availability should be regulated. This was particularly evident in response to questions on the further use of genomic data and renewed consent by those affected:

“*Well, there is always the question of what you want to achieve with it, so what sense does it make to store these data forever and ever? So for me, wouldn*'*t it make sense to send this data record, which was once collected by me, to me personally sometime, which is then somehow put on a chip in my ear and I can decide for myself who may read this chip? […] So this data record is in principle my property; I can reclaim it again sometime or request its deletion” (PSY IX 20:34)*.

In this example, the donor of the genetic sample would like to retain control over the obtained data. The participant's opinion on the further use of the data through third parties—along with the possibility of reclaiming as well as deleting the data—points to the attribution of high significance and the need to protect information in these data that concerns the self. The participants often noted such concerns about transparency and data abuse when it came to their own willingness to participate in sequencing:

“*Well, on the one hand, I don't want to know. [What] is going on has to do with my identity. And I don't want to make it totally transparent for myself and for other people. I don't see any medical benefit, either. And the third issue is that I see the risk of my data being misused. The health insurance companies want to know that. My life insurance company wants to know. […] I don't know who else wants to know everything. And that's why I don't want to do that” (Person 33, male, FG VI 26:01)*.

In the view presented here, too, one's own identity becomes tangible through genomic data, and the protection of identity represents a higher good than knowledge about oneself. From the participant's point of view, the assumed minor medical benefit of genomic information does not justify the invasion of privacy and the potential dangers of identity loss. This perspective is supported by a focus group participant who does not believe that the benefit of genomic information outweighs the danger of making her identity transparent:

“*If I want to know my ethnic origin, then I do […] genealogy. For that, I do not need to look into my genes. If I want to find out if I am pre-disposed to obesity […], I only have to look at my parents or my grandparents […]. With lactose intolerance, I find it so unimportant to know that it does not justify a genetic test for me at all. Or to know which pre-dispositions to appearance are present—I do not want to know. My personality is then lost for me. I do not want that at all. And my personality is not in the genes; I create it myself” (Person 12, female, FG II 24:04)*.

All respondents to both survey methods were thus in favor of a high standard of data protection and had reservations about the free use and exchangeability of data; they believed that the danger of transparency and disclosure of the most sensitive information justified the restriction of the use of sensitive data. Genomic information was attributed to making those affected discreditable and vulnerable, resulting in critical attitudes among participants that were reflected above all in mistrust of governmental and non-governmental organizations. These attitudes played a clear role in participants' assessment of the possibility of sequencing the entire genome:

“*It reminds me so much of [Aldous] Huxley's Brave New World, where you raise the kids with bottles and give them certain talents. And the others should only stand at a machine anyway and do some stupid work—you don't give them any cognitive skills or anything at all. And this here is on a somewhat more elaborate level” (Person 28, female, FG V 38:21)*.

Here, the participant describes fears based on Huxley's 1932 dystopian novel *Brave New World*, in which humans are conditioned to possess certain qualities and must live in castes that are suited to their skills. The quote makes clear that the participant sees human identity as something fateful that cannot be determined in advance by third parties—but genomic information is has the potential to threaten this indeterminacy. The participant sees the use of genomic information as an intervention into the openness of human destiny, using such metaphors referring to human destiny to express her suspicious attitude and fears concerning the possibilities of genomic sequencing. This use of metaphors was especially common among the focus group participants, who had a lack of personal experience as patients or study participants. In the following quotation, the participant continues to underline her mistrust:

“*They open the door to optimization. Not only physical but also mental optimization. Just think this through: You will have optimized people at some point. Yes. Those who are more intelligent, faster, more resistant. Today, people are already trying on a small scale, for example with soldiers, to physically optimize them. This will be an interesting future” (Person 28, female, FG V 39:07)*.

These assumptions show that genomic information is assessed far more in terms of its impact than merely as an information resource: From the participant's point of view, genetic knowledge not only makes people transparent, but can also optimize and improve them. At the root of this is a perspective that sees the human being as capable of being changed, adjusted and developed at will. This idea points in the direction of eugenic intentions, which, from the perspective of the interviewees, could be realized using high-throughput sequencing. Here, the danger of determination of human identity again plays an important role.

### Genomic Information as a Risk to or Confirmation of Family Identity

In the context of identity-relevant aspects of genomic information, the consideration of participant's own family and kinship is crucial. The family and kinship environment was often used by participants as a point of reference that provides information about pre-dispositions, developments and burdens related to one's own person. In the following example, the interviewee, who had previously participated in a sequencing study control group, stated that she did not wish to make use of the additional findings because she had no known pre-disposition to disease and therefore did not want to harm her family:

“*Just as it was with me now with this study, when they said, “We have incidental findings, should we communicate them to you?,” I simply said, “I do not want to know anything about incidental findings,” and I still say so. That opinion is perhaps connected with my family environment. No hereditary illnesses have occurred there. One does not talk about probabilities at all in this case” (PSY VII 16:42)*.

The interviewee wishes to protect her family's external image. Genomic information, which could offer reports about the probability of illness or random findings, may call this image into question. She then emphasizes that, on the basis of her family history without its genetic background, she assumes that nothing would be found with the help of sequencing.

Two strategies can be discerned here: the prevention of damaged identity through information avoidance and the relativization of technical possibilities, which from the participant's perspective cannot provide new insights. To reference Goffman's theory on coping with damaged identity (Goffman, 1963), the family in this example is described as not being pre-disposed genetically, but as having an identity that may be discreditable. The interviewee thus applies techniques of information control—in this case avoidance—in order not to damage the existing identity. Genomic information and additional findings are therefore relevant to identity here, since they represent a danger to the participant's and her family's identity, which is seen as the basis for their own health and positive development.

Another identity-relevant link between genomic information and kinship or family histories can be seen in the following example, which addresses a possible identity concept that is based on biological kinship along with a collective concern among generations. It comes from one of the previously-mentioned psychiatric patients, who responds to her own experience with GHS as follows:

“*I asked myself about this genome sequencing because my mother had breast cancer years ago, and you ask yourself the question, “Do I now have a higher risk myself? Do I want to know it myself or do I not want to know it and what else would appear?” In this respect, the questions always have a “self-reference” (PSY III 13:45)*.

In contrast to the previous example, this participant assumes that genomic sequencing can provide important additional information about suspicious facts from the health-related family history, which the interviewee suggests here with the term “self-reference.” Genomic information is therefore not seen here as a danger to one's own or family identity but as a confirmation or rejection of biographical narratives in relation to one's own disease-relevant risks and probabilities. Genomic information thus contributes to the formation of collective identities in families and kinships.

A similar example can be found in the account of a mother with a daughter in psychiatric treatment, both of whose genomes were sequenced. The mother describes the similarities between daughter and father by means of genetic pre-disposition and has incorporated her own interpretation of diagnostic and scientific results into her representation of the family. Moreover, according to her statement, the family history provided an argument for the examination of the genome:

“*My little daughter [is genetically] not at all [like me]. She is genetically like my husband [laughs] […]. There are neurobiological disturbances, which probably have a genetic cause but are determined by other things, and there we also had done such a genetic history [refers to genomic sequencing], because in my younger daughter the disturbance is very pronounced […] and that's what they included in the diagnosis at that time, because there were strong indications through the statements of my mother-in-law and my husband that there is such a genetic disposition” (PSY II: 12:51)*.

The relevance of identity can be seen here, on one hand, in the examination of suspicious facts based on stories from the family context. On the other hand, identity is also implied in the description of similarities within the family based on individual interpretations of genomic information. Furthermore, reference to a layered model of identity can also be established here: Biographical narratives, descriptions and interpretations of family in relation to genetic pre-dispositions can be seen as a familial layer of a person's identity. Such statements were also found among the colorectal cancer patients who were interviewed. In the following example, the possibility to learn more about familial pre-dispositions is considered to be positive:

“*That's all right if there are people who are willing to do it. It is not only about this one person, it is automatically about the family, the descendants […]. For example, my grandma and grandpa lived to be very old. They were all over 75, but probably died of cancer. That is logical. So you can assume that. My grandfather smoked and may have had lung cancer, since science was not developed enough to say that he had it. One could only assume that. You would have seen something if they had opened him up after he died. […] But who would have done that before? They were buried and that was the end of the matter” (ONK I 12:15)*.

Again, the patient's own cancer diagnosis is used as a basis for assessment. Genomic information is seen as a way of confirming family identity and verifying the assumption that cancer is part of the family history.

Genomic information represents an important area for family identity concepts, allowing identity-relevant links to family and one's own identity to be established. At the social level, the family is ascribed a high degree of identity relevance, which is compared to and evaluated against the statements offered by genomic information. This may lead to the implementation of techniques of information control in order to protect family identity. The relevance of the social and family environment is thus implied, influencing the attitudes of the interviewees in addition to individual experiences in the medical context.

### Future Life Under the Influence of Genomic Information

Many respondents saw genomic risk information as valid evidence that could have a strong impact on their own lifestyles. In the case of high disease probabilities—such as in the following example, where an 80% risk for dementia had been found—participants discussed topics of lifestyle, prevention and adapted health-oriented behavior:

“*Ok. So if I was to get this risk forecast, I think it would be a wake-up call for me that you are already doing everything you can for dementia in many ways. This starts with nutrition, up to all kinds of training” (PSY III 13:46)*.

This shows a different form of identity relevance: Through statements about pre-dispositions, genomic information can have an effect on self-image and serve as a source of information on the risks of one's own lifestyle. In a genetically pre-disposed person, this can lead to identity-relevant self-attribution. Further action can be influenced by this attribution, as shown in the above example by the proposed change in diet and focus on training. This self-attribution may or may not be permanent, but it does exert an influence on future behavior and interaction—indicating a new and active relationship to oneself and one's future, as described by Novas and Rose (2000). These attitudes are often linked to experiences that interviewees describe in their social environments. In this case, such an attitude can be traced back to various lifestyles and diets with which the interviewee became familiar in her social environment and that she associated with a direct effect on health and longevity:

“*I still have a […] grandmother of whom I am very proud, who really keeps herself really active with an interest in everyday life in the present with crossword puzzles and newspaper reading. And the sad counterexample is a friend in his mid-80*'*s who died very recently, because he had already been old in his head for 20 years. Exactly because of that, I think each human has the chance to work against their risk factors. […] Also, with cancer patients, there are often very devastating diagnoses that turn out differently in reality and that is what would give me courage. I would try to belong to these 20%” (PSY III 13:46)*.

By emphasizing social environmental factors and one's own possibilities for influence over one's health, the interviewee makes clear that despite the attributed high significance of genetic pre-disposition, its influence can be relativized:

“*That means the social environment, the thinking, the psyche but maybe also the nutrition and everything surrounding these topics, just these “soft factors,” I call them, they obviously have a much bigger influence on our life and our health status than what the genome is indicating now, and this is the opportunity for us humans” (PSY III 13:52)*.

This is a non-deterministic view of genomic information, to which some influence is attributed, but which must be seen in the context of other influencing factors; this statement contrasts with the deterministic view from the focus group quote in the first identity-relevant field. Another strategy—of consciously viewing probabilistic risk information as statistical statements that are not based on individual values, thus relativizing their significance—is also evident here:

“*Well, I wouldn't put too much weight on it, because that's just a probability, and anyway the question is, can I change this probability a bit? So I would not start any medication, since they can*'*t tell me the side effects and other consequences of the medicine because everything has consequences in some way” (PSY VI 15:37)*.

Due to their uncertainty, statements on the probability of illness are not regarded by this participant as deterministic but as one option among several. The interviewee looks critically at probability statements and emphasizes their disadvantages, claiming that he would not perform any actions based on such a statement. The following example shows that the participant's attitude can also be directly related to experiences in a medical context. The interviewee cites his high blood pressure, which has no negative effects for him and should be considered a natural deviation from the average:

“*For example, I wouldn't take any preventive medication in any direction that might delay that by a year or two. I'll give you an example: I have high blood pressure. So when I go to the cardiologist, he says, “You have to take [blood pressure medicine] and so many milligrams of it.” I don't take it because of a consultation with another doctor, because there are limits of 140 to 80. Above that limit, you have to take something, but every person is different, every person is an individual, and I say I live well with that. I don't have any risk factors, I'm not too fat, I do sports, I don't have anything that normally applies. So I don't take medicine or whatever it is, you know? Maybe I'll break down sooner, but all the side effects of the drugs may make me more likely to break” (PSY VI: 15:42)*.

In this example, individuality is emphasized as a contradiction to statistical probability statements. From the respondent's point of view, these probabilistic statements can at best offer hints or open-ended interpretations, but are not required to have a concrete effect on one's own life. In addition, the participant's strategy of relativization—by emphasizing statistical difficulties—indicates that education level also plays a major role in determining one's attitude. The extent to which genomic information is viewed as valid and whether it is taken particularly seriously or can be influenced depends on various knowledge levels and experiences in the social and medical environment.

Although the respondents emphasized that they can influence their future health through appropriate behavior, the influence of probabilistic results on their idea of future lifestyles was nevertheless evident. Their uncertainty concerned not only the validity of the information, but also the possible time of onset of the disease. In the following quotation, this uncertainty is described as a restriction to a self-determined life:

“*I can get into a deep crisis depending on the test result. I can't imagine that there are more positive things than negative things going on when people start wanting to know what might be, when, how and where. That only blocks one personally. That is again this fear in the back of one*'*s mind because these results could also be misused too much. How am I supposed to say this? It sounds so pathetic now, but that is no longer a self-determined life, in a certain way. You can't hide it, depending on the results. It is somehow no longer a self-determined life, one is too restricted or one thinks too much about these things” (PSY VII 16:46)*.

The possible psychological burdens—as well as the protection of self-determination from restrictions based on genomic risk information associated with uncertainty—led the participant to waive the disclosure of certain results. In his view, genomic information has the power to impair the prior self-image of a healthy person as well as their entire life, because the self-image that assigns the status of “healthy” or “ill” also influences the further health-relevant actions of the person concerned. The disclosure of additional findings or general feedback is therefore rejected by the participant. In addition, this example shows that mentally ill people may also use their experience of illness as a basis for assessing the handling of genomic information.

Despite the evidence showing that genomic information has a great deal of identity relevance, fluctuations in identity relevance among the participants can also be identified, clearly pointing in the direction of a multi-layered concept of identity over time (Zeiler, 2007); high expressiveness was attributed only to certain stages of life and situations. With a view to an approaching end of life, genomic information and additional findings from sequencing procedures seemed to lose importance for the interviewees:

“*Right, so I think that it depends on which areas, regions and it may well be at which age I am, because there is now the 30-year-old woman. If I get such a prognosis as an 80-year-old I would actually deal with it quite differently than this 30-year-old will probably deal with it. So I can already imagine that the older ones will just say, ok, I've lived my life pretty well. And such a 30-year-old still has a lot to do [laughs]” (PSY IX 20:31)*.

This is based on the assumption that older people who are at or near the end of their lives are no longer afraid of diseases or incriminating health information. This participant, however, is speaking about a distant future that is not yet a concrete reality. With regard to identity relevance, this statement shows that the significance of information and results is influenced by individual circumstances, such as advanced age or the approaching end of life. The identity relevance of genomic information can thus be influenced by biographical fluctuations and can be regarded as context-dependent.

### Identity Benefits Through New Possibilities of Legitimation

Despite a dominant skeptical-to-negative view of the use of genomic information, positive attitudes based on personal experience can also be identified in the material. Genomic information was seen by some as helpful, because it offered identity gains based on legitimation possibilities that would not otherwise have existed. This is illustrated by the following quotation from the aforementioned mother, who describes her experience with the application of high-throughput technology in her daughter's diagnosis:

“*In this respect, this was also done back then to give my daughter a little something to hold on to and to awaken in her the awareness that many things that happen to her are simply also,… yes, so to speak, due to illness. Because she always said, “Why can't I perceive it like other people, why can't I behave correctly?,” so that's a question that came to the child very early” (PSY II 12:51)*.

The interviewee describes the aim of the testing, namely to awaken a certain awareness in her daughter by identifying the cause of the disease and her divergent behavior. The disease can thus be legitimized and its genetic basis seen as part of the daughter's identity. In the end, this can lead to the creation of a patient identity based on genomic information. The interviewee then explains that sequencing was the last possibility for identifying the causes of her daughter's unspecified mental illness, representing the end of a long odyssey in the field of medical diagnostics:

“*And we also found it helpful to know that, because then we can adjust our lives to simply structure certain things and it also relieves us of the responsibility to say that we are total failures in parenting and that there is something socially wrong with us. At that time, it also totally relieved us as a family. So far, the experiences we have had personally are only very positive” (PSY II 12:7)*.

What is important for the interviewee is that because the disease and divergent behavior of the daughter have a genetic cause, she no longer needs to be seen as an ineffective mother. The results of the sequencing have thus created a basis of legitimacy that prevents the family from being stigmatized as a negative environment for the daughter. Here, the conscious use of genomic information plays an important role in public image. The results of the genome sequencing have high identity relevance for the family, which is reflected in the relieving function against negative attributions of third parties; this also represents an identity gain. The self-attribution as genetically pre-disposed to psychiatric disease makes apparent the social self-location and self-relation (Mead, 1934; Keupp, 2018) to genomic information mentioned in the theoretical part of this paper. This social self-location serves as a fit between the subjective inside and social outside of the participant and it fulfills the purpose of legitimation. Furthermore, this example demonstrates that in addition to the participants' dominant attitudes based on rejection of GHS, the material also shows open-minded and supportive attitudes grounded in positive experiences, underlining the relevance of experience and knowledge.

## Discussion and Outlook

In view of the results, it is difficult to say that new social identities can only be created through the establishment of new technologies and broader diagnostic possibilities; otherwise this could be understood as technological reductionism (Atkinson et al., 2006) and an excessive lowering of complexity in the context of identity emergence. Nevertheless, the results show that genomic information has an important influence on the construction of identity. Two basic concepts of identity can be discerned in the everyday understanding of the interviewees:

The first concept of identity is based on deterministic and exceptionalistic views. From the interviewees' perspective, identities or parts thereof can be made visible via information and data. This notion led the participants to emphasize the protection of genomic data in order to prevent permanent discrediting and damage to identity. The handling of genomic information affects both self-determination and privacy, which from the respondents' point of view must be preserved, leading to negative attitudes to the handling of genomic information. These attitudes were influenced by concerns about mere loss of data as well as an increased risk of third-party intrusion into privacy. The participants expressed not only the fear of losing control and independence (Wüstner, 2002) but also of making visible their complete, previously hidden identity-relevant information. This also helps to explain the respondents' varying levels of agreement to providing genomic data for research purposes, depending on whether the research is for profit-oriented companies or publicly funded institutions (Mählmann et al., 2016). The attitude that genomic data belong to their donor has already been addressed in other studies (Townsend et al., 2012) and is becoming increasingly important, for example, in discussions concerning “big data” (i.e., the collection, analysis and linking of a wide variety of data, such as self-collected lifestyle data with medical data). In the deterministic attributions presented in the first identity concept, the topic of the ownership of genomic information represents an important facet of this “big data” discussion; there are, however, major reservations on the ethical-scientific side. The German Ethics Council criticizes the concept of ownership of genomic data, referring in its opinion to data sovereignty based on informational freedom (in accordance with informational self-determination). This criticism is intended to enable those affected by GHS to intervene in—but not stop—data flow on the basis of personal preference; it is also in line with societal requirements for the use of data in the sense of solidarity and justice (Deutscher Ethikrat, 2018).

In contrast, the non-deterministic identity concept, which emphasizes the relativity of genomic (risk) information and relies on the influence of external factors, shows above all a biologically undetermined self-perception. In this view, actions, intentions and attitudes play an important role in future personal and health development; identity cannot be expressed or even stored in figures, data and information. The wide range of deterministic and non-deterministic concepts of identity— described by Chadwick (2003), in the context of genetic information, as opposite to one another—can thus also be found in the framework of genomic information. Between these concepts are fluid transitions, and it is often possible to show them being used by one and the same person. They are expressed, however, in different contexts: When describing negative attitudes due to worries and fears, the use of the first (deterministic) view is more evident, while the central statements of the second (non-deterministic) concept occur primarily in the imagination of own personal affection in connection with risk information or additional findings. This is not, it should be noted, a fixed dichotomy, since shared attitudes between participants can also be identified; for example, the interview and focus group participants have a shared fundamental distrust of the use and dissemination of sensitive information.

An identity concept based primarily on the influence of genomic risk information was most often reflected by participants in their discussion of uncertainty and fears in connection with probabilistic statements. Here, as in the area of genetic testing, the impairment of individual psychological well-being, self-image, personal life plan, social relationships and quality of life were addressed. Self-references were established, and personal experiences from medical and everyday contexts were used to assess the influence of genomic risk information (Kollek and Lemke, 2008).

It is unclear to what extent a duty toward future-oriented action around health is represented in the corresponding perspectives to probabilistic statements. Both viewpoints can be found in the material: on the one hand, a focus in current action on future development, and on the other, the rejection of certain actions (e.g., taking medication), despite indications based on genomic information that they would be helpful. The assumption or rejection of a risk identity (”person at risk“) (Novas and Rose, 2000) seems to depend on the respective use of a deterministic or open identity concept, which in turn is based on educational background, knowledge, and experiences in the social and medical context.

The interviewees' statements primarily used an everyday psychological understanding of identity as a subjective and isolated element, which at first glance seems to correspond to the psychological interpretation of personality. As described in the theoretical section of this paper, however, Mead (1934) and Giddens (1991) offered a meaningful distinction between the concepts of identity, which in a reflexive process refers more to attributions; self-labeling (self-attribution); and negotiation; these were identifiable in the relevant quotes above. The concept of identity is therefore also to be distinguished from the psychological concept of personality, which traditionally derives from a different research discipline (Haller and Müller, 2006). Nevertheless, foreign and self-attributions play an important role for identity construction, especially in biographical narratives and attitudes to life under the influence of genomic information. In the confirmation or rejection of health-related family and biographical narratives, family attributions of personal development are an especially important source for one's own self-image; the interviewees often mentioned issues that are considered inherited by the family.

Empirically, some evidence for the ”multi-layered concept of identity“ developed by Zeiler (2007) was found. This became particularly clear in the reference to the age at which a person receives genomic information, which can lose or gain in relevance over time. The multi-layered concept is also generally reflected in the explicit attribution of lower identity relevance of genomic information, which is particularly evident among non-deterministic viewpoints (compare the examples in the fourth topic area): Here, identity is more than mere information and bare numbers. It is associated by respondents in everyday understanding with personality, or what Mead calls ”consciousness.“ The underlying concept of identity, however, here refers to attributions and self-attributions that contribute closely to the construction process of identity in the context of interaction and social action and are essential for the concept of identity in the sociological sense. The layer model described can also be reconciled with this, since the aforementioned ascriptions and self-attributions are aimed at specific layers of identity.

The deterministic concept of identity, which was exemplified by the quotations from the first topic area, cannot be reconciled with a layer model, since here no ”degrees of difference“ (Zeiler, 2007, p. 27), and thus no individual layers, are granted. As a result, in the deterministic view, an identity based on genomic information must always remain the same over time (2007). Further research is needed here, however, as this is a purely philosophical-theoretical concept that must be investigated via empirical study.

The statements of the interviewees demonstrate that under the influence of genomic information, people enter into new and active relationships with themselves and their own futures (Novas and Rose, 2000), at least in the sense that the respondents wished to orient their health-relevant actions to genomic information. Since, however, here is no evidence to suggest that long-term lifestyle changes are made based on genomic information, such a sustainable effect of genomic information tends to be viewed critically (Hollands et al., 2016; Lindor et al., 2017).

The limits of the study presented here lie in the chosen qualitative study design: These are results of interpretative social research without statistical representation. Nevertheless, this approach is important, since an iterative and explorative development of the field can serve to open the door to further research projects.

Of course, the results must also be seen in light of the limitations of the sample. The heterogeneous survey structure with regard to experiences with GHS should be noted: Persons who had participated in sequencing had a low knowledge level about current problems in dealing with genomic information. Due to little options for selection of ongoing studies at the time of recruitment, the majority of those personally affected were located in the psychiatric sector. As GHS is a relatively new technology, there is not yet a high degree of implementation in clinical practice outside university clinics. Against the background of the actuality of the social scientific and ethical questions, the interviews and focus groups nevertheless allowed for the expression of a certain range of current attitudes and subjective opinions regarding the handling of GHS and genomic information. The negotiation of an identity (Giddens, 1991) between two individuals under the influence of genomic information could not be shown on the basis of the material; methods of (non-)participating observation would certainly be more suitable for this purpose. Still, it was possible to identify attributions and self-attributions that could manifest during the construction process of identity in the context of interaction.

For further research, it would be useful to investigate the age-specific relevance of genomic information on an appropriate sample of people in old age. The loss of identity due to the availability of sensitive information remains a hypothetical matter, as it can largely be found in the interviewees' descriptions of their dangers, worries and fears. In contrast, the added value of GHS to identity was identifiable through statements based on concrete experiences. Both aspects must be differentiated on the basis of further empirical examples. Overall, however, a rough framework for further analyses in this field was created via the identity-relevant topic areas.

The combination of the different patient groups in this work also represents further limitations that should be emphasized here. As previously described, both psychiatric and colorectal cancer patients used their experiences in the specific medical-therapeutic context to determine their attitudes and evaluations regarding the handling of genomic information. This study indicates that patients with life-threatening colorectal cancer may have a different perspective on the problem; their disease seems to have a very strong impact on the self-understanding and identity relevance of genomic information. In this sense, they are affected differently than psychiatric patients, who also draw on their experiences but do not suffer from an acutely life-threatening disease (although, for a few psychiatric diseases, this possibility may exist). The fact that a psychiatric disease has a different influence on the construction of identity than cancer should be re-emphasized. Accordingly, the degree of affectedness by genomic information is to be evaluated differently for all groups of respondents. How exactly it is to be assessed remains the task of further research.

Overall, it should be underscored that there are no significant differences between the two groups of respondents regarding the use of the described identity concepts. The contrast in the interpretation of the material refers primarily to identity-relevant aspects of genomic information, which is reflected in the five thematic fields and, finally, in the deterministic and non-deterministic concepts of identity. For this reason, the article and the evaluation were based on an analytical combination of respondent groups rather than simply contrasting laypersons with patients. This was not and is not the subject of the article, since the primary concern was the identification of identity-relevant aspects in dealing with genomic information. Here it should again be made clear that it is, of course, also possible and important to distinguish between the respondent groups with respect to their evaluation of and attitudes toward genomic information. This distinction obviously results from the participants' respective experiences in the specific medical-therapeutic and everyday contexts, both of the psychiatric and colorectal cancer patients and of the interviewed lay people as representatives of the public. Nevertheless, the question of more detailed experience reports from those personally affected in the field of GHS remains open. People who have consciously opted for GHS in order, for example, to gain appropriate knowledge in the case of disease without a clear diagnosis would be of great interest here, above all in the field of clinical-medical application. In addition, the area of direct-to-consumer genetic testing (i.e., making genetic and genomic information available to end consumers without the direct participation of physicians in genetic counseling) remains an extremely exciting area for researching identity-relevant influences outside the medical context. Researchers should also examine how genome research is to be classified as part of the various discussions on the transformation of society: First considerations are presented here by Reckwitz (2017), who notes a turn to the particular of each individual case on the basis of genome analysis as a form of digitization, as well as by Mau (2017), who describes the self-measurement and quantification of the social as sociometry.

Taken as a whole, the thematic fields explored in the material show that genomic information is highly relevant to identity and can be overestimated by respondents under certain conditions and depending on the influence of educational background, experience and knowledge. In the pediatric field, this has already been shown in a qualitative study of 51 parents and patients by McGowan et al. (2013), which focuses on those personally affected and on laypersons who cannot be characterized as enthusiastic about collecting information about their own genetic profiles. Based on the results presented, however, it can be concluded that unrealistic expectations and attributions speak in favor of improving the teaching of new possibilities for genetic diagnostic procedures.

This study confirms that generally shared views in dealing with genomic information are based on outdated discourses and, despite the described change in dealing with complexity of information, the lay discourse is still strongly influenced by deterministic and exceptionalist views (Zwart, 2007). The deterministic views of the participants can be traced back, for example, to debates on locating the causes of cancer in the individual (Wolf, 2001); public reporting on genetic engineering (Görke et al., 2000); and the euphoria of the human genome project, in which the book of life was believed to have been decoded by creating a human reference genome (Lemke, 2002). The complexity of genomic correlations emphasized in current debates, which has by no means yet been fully understood, was only partly taken into account by the participants, and therefore an essential factor of genomic information has only briefly been considered thus far. Particularly in the focus group discussions, the ”shift from genetic determinism to the understanding of complexity“ attested in the scientific field by Zwart (2007) cannot be identified. Nevertheless, some statements emphasized the more comprehensive character of genomic information and associated it much more strongly with one's own identity.

Finally, it should again be stressed that, due to misinterpretation and misunderstanding, the deterministic attributions described here can ultimately lead to disadvantageous decisions, and that processes of informed consent and medically relevant action may be impaired. Thus far, this problem could also be identified in the context of the benefit analysis of GHS on the basis of false assumptions about the validity of genomic tests in personally affected people (Urban and Schweda, 2018). Still, questions related to identity development under the influence of genomic information—along with the expansion of existing theoretical approaches against the background of further establishing genomic sequencing technologies—remain an important topic in the field of medical sociological research.

## Data Availability Statement

The datasets generated for this study will not be made publicly available. The data from the interviews and focus groups (transcripts) belong to the Department of Medical Ethics and History of Medicine, University Medicine Center Göttingen (Prof. Dr. Silke Schicktanz).

## Ethics Statement

The studies involving human participants were reviewed and approved by Ethics Committee University Medical Center Göttingen (Ethics Proposal #16/10/14). The patients/participants provided their written informed consent to participate in this study.

## Author Contributions

All essential work of the presented study was done by AU. Persons who, according to the criteria for authorship, provided support and assistance with the practical implementation of the study or helped with the revision of the manuscript are listed in the Acknowledgments section.

## Conflict of Interest

The author declares that the research was conducted in the absence of any commercial or financial relationships that could be construed as a potential conflict of interest.

## Footnotes

^1^This article uses the following definition: Additional findings are not related to the initial question, but can still be relevant for the health or family planning of the examined person or their relatives (GFH, 2013; Rudnik-Schöneborn et al., 2014).
